# The role of succinate and ROS in reperfusion injury – A critical appraisal

**DOI:** 10.1016/j.yjmcc.2017.06.016

**Published:** 2017-09

**Authors:** Tatyana N. Andrienko, Philippe Pasdois, Gonçalo C. Pereira, Matthew J. Ovens, Andrew P. Halestrap

**Affiliations:** School of Biochemistry and The Bristol Heart Institute, Medical Sciences Building, University of Bristol, Bristol BS8 1TD, UK

**Keywords:** 5-cH_2_DCFDA, 5-carboxy-2′,7′dichlorodihydrofluorescein diacetate, ANT, adenine nucleotide translocase, Bad, Bcl-2-associated death promoter, Bak, Bcl-2 homologous antagonist/killer, Bax, Bcl-2-like protein 4, Bcl-2, B cell lymphoma 2, BCECF, 2′,7′-*bis*-(2-carboxyethyl)-5-(and-6)-carboxyfluorescein, Bcl-xL, B-cell lymphoma-extra large, Bid, BH3 interacting-domain death agonist, CK, creatine kinase, CrP, creatine phosphate, CsA, cyclosporine A, Cyp-D, cyclophilin D, DHE, dihydroethidium, Drp1, dynamin-related protein 1, G-6-P, glucose-6-phosphate, GSK3β, glycogen synthase kinase 3β, HK, hexokinase, IF1, ATP synthase inhibitor factor 1, IMM, inner mitochondrial membrane, IP, ischemic preconditioning, IR, ischemia/reperfusion, Mdivi-1, mitochondrial division inhibitor 1, LS, light scattering, MCU, mitochondrial calcium uniporter, MitoPY1, Mitochondria Peroxy-Yellow 1, mPTP, mitochondrial permeability transition pore, NCLX, Na^+^/Ca^2+^ exchanger, OMM, outer mitochondrial membrane, PCr, phosphocreatine, PKCε, protein kinase Cε, pmf, proton motive force, PO1, Peroxy-Orange 1, REF, reverse electron flow, ROS, reactive oxygen species, SOD, superoxide dismutase, VDAC, voltage-dependent anion channel, Succinate, Ischemia/reperfusion injury, Heart, Mitochondria, Reactive oxygen species, Calcium, Permeability transition pore, Hexokinase

## Abstract

We critically assess the proposal that succinate-fuelled reverse electron flow (REF) drives mitochondrial matrix superoxide production from Complex I early in reperfusion, thus acting as a key mediator of ischemia/reperfusion (IR) injury. Real-time surface fluorescence measurements of NAD(P)H and flavoprotein redox state suggest that conditions are unfavourable for REF during early reperfusion. Furthermore, rapid loss of succinate accumulated during ischemia can be explained by its efflux rather than oxidation. Moreover, succinate accumulation during ischemia is not attenuated by ischemic preconditioning (IP) despite powerful cardioprotection. In addition, measurement of intracellular reactive oxygen species (ROS) during reperfusion using surface fluorescence and mitochondrial aconitase activity detected major increases in ROS only after mitochondrial permeability transition pore (mPTP) opening was first detected. We conclude that mPTP opening is probably triggered initially by factors other than ROS, including increased mitochondrial [Ca^2+^]. However, IP only attenuates [Ca^2+^] increases later in reperfusion, again after initial mPTP opening, implying that IP regulates mPTP opening through additional mechanisms. One such is mitochondria-bound hexokinase 2 (HK2) which dissociates from mitochondria during ischemia in control hearts but not those subject to IP. Indeed, there is a strong correlation between the extent of HK2 loss from mitochondria during ischemia and infarct size on subsequent reperfusion. Mechanisms linking HK2 dissociation to mPTP sensitisation remain to be fully established but several related processes have been implicated including VDAC1 oligomerisation, the stability of contact sites between the inner and outer membranes, cristae morphology, Bcl-2 family members and mitochondrial fission proteins such as Drp1.

## Highlights

• Mitochondrial ROS production during early reperfusion (RPF) occurs after mPTP opening.
• Conditions do not favour ROS production by reverse electron flow during early RPF.
• Ischemic preconditioning (IP) does not attenuate succinate accumulation in ischemia.
• IP reduction of ROS and Ca^2+^ during RPF is secondary to attenuation of mPTP opening.
• IP attenuates mPTP opening by mechanisms independent of ROS and Ca^2+^ such as HK2.

## Graphical abstract

**Figure:**
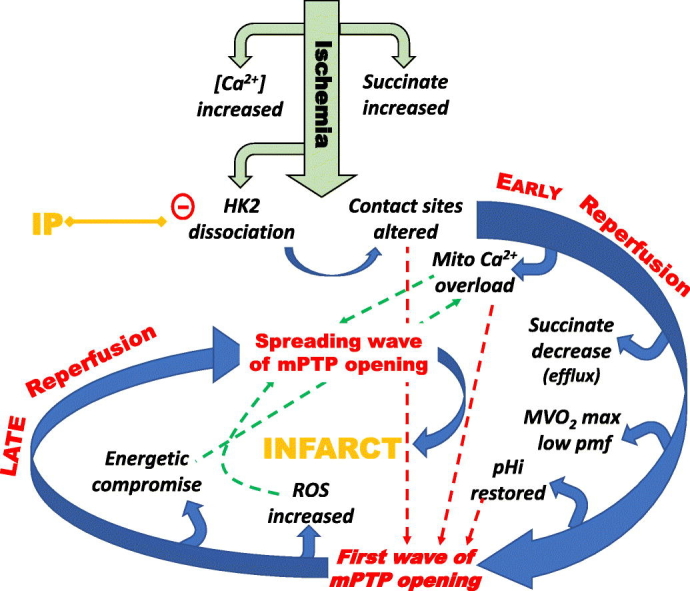

## 1 Introduction

It is now widely accepted that the irreversible injury of the heart which occurs during reperfusion after a prolonged period of ischemia is mediated, at least in part, by opening of the mitochondrial permeability transition pore (mPTP) [1], [2]. This starts after about 2 min of reperfusion [3], [4] and coincides with the intracellular pH (pHi) returning to pre-ischemic values from the low values (< 6.5) that are reached after prolonged ischemia and which inhibit mPTP opening [5]. Opening of the mPTP not only compromises cellular bioenergetics, thus impairing the re-establishment of pre-ischemic levels of ions such as calcium, but also causes mitochondria to release reactive oxygen species (ROS) [6], [7]. The resulting increases in ROS and [Ca^2+^] induce further mPTP opening and bioenergetic compromise leading to a spreading wave of necrotic cell death that produces the infarct [1], [8]. Pharmacological inhibition of mPTP opening by cyclosporin A (CsA) [9], sanglifehrin A [10] or cinnamic anilides [11] can protect hearts from ischemia/reperfusion (IR) injury as can ischemic preconditioning (IP) and post-conditioning, both of which also inhibit mPTP opening [1], [8].

Although the precise molecular composition of the mPTP remains uncertain [2], [12], [13], it is well established that increases in matrix [Ca^2 +^] trigger mPTP opening and that its sensitivity to [Ca^2+^] is greatly enhanced by oxidative stress, elevated phosphate and decreased matrix adenine nucleotides [1]. These are all conditions associated with IR. Indeed, the combination of increased matrix [Ca^2+^] with oxidative stress is now widely regarded as the main trigger for mPTP opening during early reperfusion [1], [2]. Oxidative stress within the matrix is an especially attractive candidate since this overcomes the powerful inhibitory effect of adenine nucleotides on mPTP opening leading to a profound increase in Ca^2+^ sensitivity [14]. Furthermore, several studies, including our own, have shown oxidative stress to increase during reperfusion and to be greatly attenuated by a variety of protocols that induce cardioprotection including IP [15], [16], temperature preconditioning (TP) [17], urocortin [18], apomorphine [19], ischemic post-conditioning [20] and sequential exposure to isoproterenol and adenosine [21]. However, it is also well established that increased superoxide production can occur as a consequence of mPTP opening [6], [7], caused in part by the loss of cytochrome *c* and NADPH from the mitochondria, both of which are important for ROS scavenging [6], [22]. Thus, it is important to establish whether increased levels of ROS precede mPTP opening during early reperfusion or occur later as a consequence of mPTP opening.

Recently, Murphy, Krieg and colleagues have presented extensive data to implicate superoxide production from the matrix surface of Complex I early in reperfusion as a key player in IR injury [23], [24], [25], [26]. They propose that this superoxide production occurs because succinate accumulates in the heart during ischemia and is rapidly oxidised by reverse electron flow (REF) at the start of reperfusion. This induces a highly reduced state of the ubiquinone binding site on the matrix face of Complex I that drives superoxide production [23]. Here we critically assess the role of succinate-mediated superoxide production from Complex I in IR injury and conclude that it is unlikely to be the primary trigger of mPTP opening in the early phase of reperfusion and which is modulated by IP. Rather, we suggest that it is elevated [Ca^2+^] that initiates mPTP opens on reperfusion and that IP attenuates other factors that sensitise the mPTP to [Ca^2+^], including the well-established dissociation of hexokinase 2 (HK2) from its mitochondrial binding site that occurs during ischemia [27], [28], [29]. However, substantial ROS production does occur later in reperfusion as a consequence of initial mPTP opening, and this leads to further pore opening and an expanding area of necrotic cell death that forms the infarct. Cardioprotective protocols such as IP prevent HK2 loss from mitochondria during ischemia and so prevent both phases of mPTP opening.

## 2 Does mitochondrial superoxide production precede mPTP opening during reperfusion?

### 2.1 ROS measurements

The American Heart Association has recently published a “Scientific Statement” on the measurement of ROS species which provides a comprehensive review of the available methods, their limitations and what combined approaches are recommended for particular situations [30]. As this article makes abundantly clear, measurement of ROS species is not straight forward, and although many different methods can be used, each approach is fraught with potential pitfalls for the unwary. Some of these issues are noted in the discussion below, but the main focus of this section is to provide a critical review of the data relating the time course of ROS formation in the ischemic/reperfused heart to the time course of mPTP opening.

#### 2.1.1 Studies using isolated cardiac myocytes

Studies using isolated adult cardiac myocytes subject to simulated ischemia and reperfusion have provided evidence that ROS production precedes mPTP opening and cell death [23], [31], [32], [33]. However, to simulate ischemia, these studies employed bicarbonate-free media and anoxia together with low pH, with or without the addition of l-lactate, followed by return to normal medium (still bicarbonate free) to mimic reperfusion. In such studies, the cardiomyocytes are usually quiescent or at best stimulated to beat at very low frequency and it is questionable whether these conditions adequately reproduce those occurring in the intact ischemic/reperfused heart. In the beating perfused heart there will be a much higher metabolic turnover and Ca^2+^ cycling rates than in isolated cardiac myocytes with the consequence that mitochondria will be in a different redox and bioenergetic state. This may reduce both their ability to accumulate Ca^2+^ and produce ROS. Furthermore, the concentration of myocytes in the heart, and their complex interactions with each other and endothelial cells, cannot be adequately reproduced when using isolated myocytes for fluorescence microscopy. Nor can the build-up and subsequent washout of metabolites that occurs in the ischemic reperfused heart, while the absence of bicarbonate will disrupt normal pH regulatory mechanisms. In addition, the studies with cardiac myocytes [32], [33] generally used much longer periods of simulated ischemia than are routinely employed in studies with the perfused heart and thus are difficult to compare with data obtained using the ischemic/reperfused heart. A more recent study using dihydroethidium (DHE) as the superoxide-sensitive dye has measured ROS production after 1–2 min of simulated reperfusion [23], but major concerns have been expressed over the use of DHE as a ROS probe, both in terms of whether the fluorescent species monitored really detects superoxide [34] and its sensitivity to changes in mitochondrial membrane potential [35] as discussed further below (Section 2.1.2). For these reasons, we suggest that any conclusions drawn from isolated cardiomyocyte experiments must be treated with caution, especially where they differ those obtained using the perfused heart.

#### 2.1.2 Studies using the perfused heart

Measurements of ROS production/oxidative stress have been made in the ischemic/reperfused heart, but the majority of these studies lacked the time resolution to establish whether ROS increases before or after mPTP opening. Thus determinations of protein carbonylation [15], [16], [17], [19], [20], [21], [24], protein thiolation [36], lipid peroxidation [37], [38], [39] and aconitase activity [23] have all clearly demonstrated that oxidative stress does occur during reperfusion, but do not provide information on whether there is a significant increase in ROS within the first minute or two, prior to initial mPTP opening. The same is true for the measurement of mitochondrial superoxide using the mitochondrial targeted hydrogen peroxide probe, mito B, assayed using mass spectrometry of freeze clamped tissue. Here the earliest time point reported was at 15 min [23], [24]. More rapid detection of increased ROS production has been obtained using hearts perfused with the electron paramagnetic resonance (EPR) free radical probe 5,5-dimethyl-1-pyroline-n-oxide (DMPO) where peak concentrations of radical formation were detected after 10–20 s of reperfusion followed by a gradual decline over the next 1–2 min [40]. It was demonstrated that these radicals were derived from superoxide since reperfusion in the presence of enzymatically active recombinant human superoxide dismutase (SOD) markedly reduced the formation of the relevant EPR signals while inactive SOD had no effect. However, the effect of SOD implies that the location of the superoxide detected was extracellular since otherwise it would not be attenuated by membrane-impermeable SOD. In a separate set of experiments in which hearts were rapidly freeze-clamped and endogenous radicals measured using EPR, a similar time course of radical formation was detected [41] which is more likely to reflect intracellular superoxide formation over this time period. Indeed, measurements of the reversible glutathionylation of glyceraldehyde 3 phosphate dehydrogenase did detect an increase within 1 min of reperfusion after 30 min ischemia [42]. The luminescent probe lucigenin (*N*,*N*′-dimethyl-9,9′-biacridinium dinitrate) also detected increases in superoxide within a minute or so of reperfusion, but these only reached a peak at about 5 min [35] which is after mPTP opening [3], [4]. It has been suggested that the positive charge on this probe may lead to its preferential uptake and retention by mitochondria and some evidence for this has been presented [35]. However, others have argued that its permeability into cells is an issue and that a significant proportion of the signal may represent extracellular superoxide production [30].

Surface fluorescence of the Langendorff-perfused heart has also been used to monitor ROS production in real time during IR. There are significant problems associated with this approach including motion artefacts, internal filtering of the incident and emitted light by cytochromes, flavoproteins and myoglobin, and autofluorescence by NAD(P)H and flavoproteins. We have discussed these issues more fully elsewhere [7]. Of particular concern is that both autofluorescence and internal filtering change dramatically in the transition between normoxia and ischemia as myoglobin becomes deoxygenated and the cytochromes and flavoproteins become reduced. Although these effects rapidly reverse on reperfusion, it is only when this process is complete that reliable conclusions can be drawn from the crude fluorescent signals. This is a particular problem for dihydroethidium (DHE) which has been employed as a superoxide-specific fluorescent probe in several perfused heart studies [35], [43], [44], [45], [46], [47], [48], [49], [50]. DHE itself is not fluorescent but once oxidised by superoxide to 2-hydoxyethidium it fluoresces at > 600 nm when excited at ~ 535 nm. Unfortunately, this makes the probe especially sensitive to changes in myoglobin oxygenation and the redox state of cytochromes. Indeed, such effects probably account for the very rapid increase and decrease in fluorescence seen upon ischemia and reperfusion respectively [43]. This makes it very difficult to assess whether rapid changes in ROS are occurring early in reperfusion. Additional concerns have been expressed over the use of DHE as a ROS probe, both in terms of whether the fluorescent species monitored reflects superoxide [30], [34] and its sensitivity to changes in mitochondrial membrane potential [35]. Nevertheless, in the perfused heart slower increases in signal are seen after about 3–4 min of reperfusion that are prevented by IP [43] and would seem likely to reflect changes in ROS as revealed by our own studies with alternative probes described below.

**Fig. 1:**
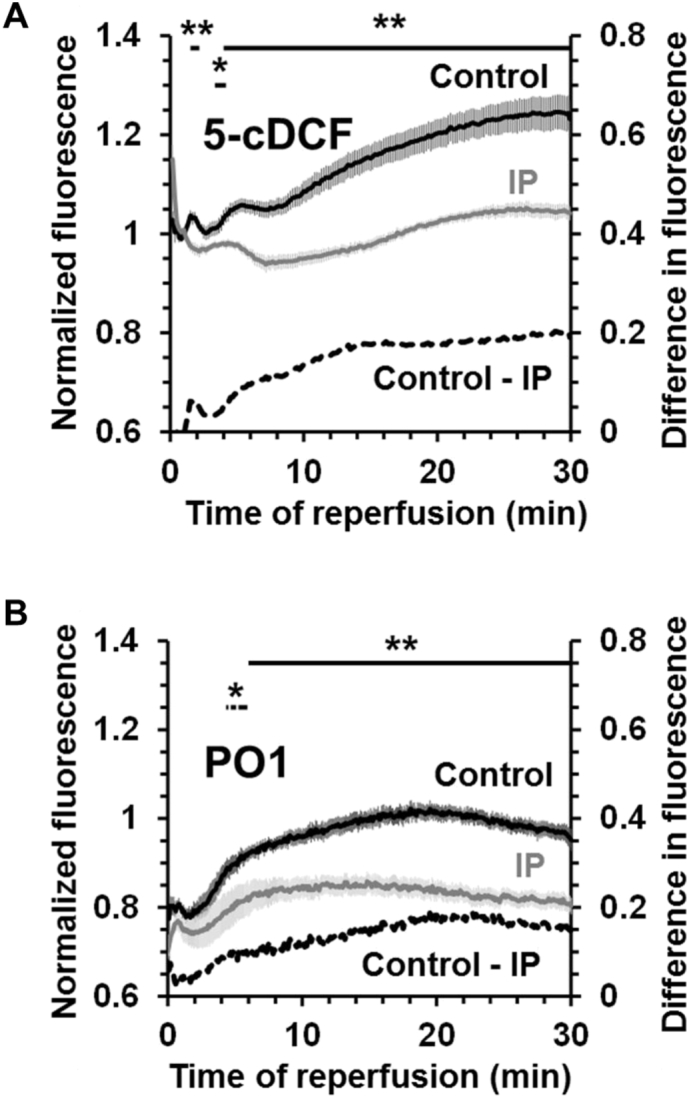
Time course of ROS production measured using surface fluorescence in the Langendorff-perfused heart subject to ischemia and reperfusion. Both control and IP hearts were pre-loaded with either 5-cH_2_DCFDA as its di-(acetoxymethyl ester) or PO1 and then subject to 30 min index ischemia followed by reperfusion for the time shown. Data are expressed as means ± SEM (error bars) for 6 (5-cH_2_DCFDA) or 9–11 (PO1) separate heart perfusions and are taken from [7] where further details may be found. Significant differences between control and IP hearts, calculated by unpaired *t*-test, are shown by horizontal lines (*p < 0.05; **p < 0.01).

We have recently published extensive data from experiments in which we continuously measured ROS production in the beating heart subject to IR using a bespoke surface fluorescence apparatus to monitor the surface fluorescence at multiple wavelengths with high time resolution [7]. Simultaneous measurements were made of hemodynamic function together with surface reflectances at both the excitation and emission wavelengths of all probes used. The latter measurements allowed monitoring of potential artefacts caused by changes in surface geometry (such as occurs during ischemic contracture), although in the optical configuration employed it was found that such artefacts were minimal. We also determined the fluorescence emission spectra for each dye loaded within the heart at various times of ischemia and reperfusion to assess the effects of internal filtering caused by myoglobin, cytochromes and flavoproteins. These measurements revealed that all fluorescent probes were likely to be subject to interference by internal filtering during the first 10–15 s of ischemia and reperfusion, although the use of the ROS-insensitive dye, calcein, monitored under the same conditions, allowed some estimate of these effects to be made. Measurements after 10–15 s of reperfusion were not subject to major artefacts and thus provided robust data. Three different ROS probes were employed to detect changes during reperfusion in control hearts and those subject to IP. These were 5-carboxy-2′,7′-dichlorodihydrofluorescein diacetate (5-cH_2_DCFDA), a generic ROS probe whose exact specificity is uncertain [51], and two caged boronate probes that are specific for hydrogen peroxide: Peroxy-Orange 1 (PO1) which measures primarily cytosolic hydrogen peroxide and Mitochondria Peroxy-Yellow 1 (MitoPY1) that is targeted to mitochondria. In parallel we measured aconitase activity in mitochondria isolated after 90 s reperfusion as an additional indicator of mitochondrial ROS. As shown in Fig. 1, both 5-cH_2_DCFDA and PO1 revealed a similar pattern of ROS production; in both cases no significant increase in ROS was detected until after about 1.5–2 min of reperfusion. By measuring the loss of calcein entrapped within the mitochondria we confirmed that this was after mPTP opening had occurred [7]. The progressive increase in ROS observed after about 2 min of reperfusion was attenuated by both IP and CsA which also reduced infarct size and inhibited mPTP opening as detected previously using mitochondrial 2-deoxyglucose entrapment [52] and more recently with calcein entrapment [7]. Thus our data with both 5-cH_2_DCFDA and PO1 suggest that these probes only detected ROS production after initial mPTP opening. We were also unable to detect any increase in mitochondrial ROS at 90 s of reperfusion using either the mitochondria-targeted hydrogen peroxide probe MitoPY1 or aconitase activity measurements [7]. As noted before, both aconitase activity and the superoxide probe mitoB did detect an increase in mitochondrial ROS after 15 min reperfusion [23], but again this is after mPTP opening and thus may well be a consequence of mPTP opening rather than a primary cause.

Overall, we suggest that ROS probes such as DHE, DMPO and lucigenin that report increases in ROS levels within the first minute of reperfusion are probably not detecting ROS within the mitochondria. As such they provide no strong evidence for IP-sensitive increases in superoxide within the mitochondrial matrix early in reperfusion that could stimulate mPTP opening. It may well be that some of these probes are responding to either extracellular or cytosolic superoxide produced by enzymes such as NADPH oxidase, uncoupled nitric oxide synthase, monoamine oxidase and xanthine oxidase [53], [54], [55], [56]. Cytosolic superoxide is likely to be rapidly scavenged by SOD, glutathione peroxidase and glutathione reductase [57], [58] before it can enter the mitochondrial matrix as suggested by the rapid increase in glutathionylated proteins such as glyceraldehyde 3 phosphate dehydrogenase [42].

### 2.2 Effects of ROS scavengers

If an increase in mitochondrial superoxide production in the matrix is a key trigger for mPTP opening early in reperfusion, then intramitochondrial ROS scavengers (reviewed in [59]) should be cardioprotective. Mitochondria-targeted Szeto-Schiller peptides (also known as Bendavia) do reduce ROS production by isolated mitochondria [60] and are mildly cardioprotective in some models of IR injury [58], [60], [61], [62], [63], [64]. However, exactly how these agents work is unclear since they do not act as scavengers of defined reactive oxygen species produced *in vitro* [60] and thus are not acting as true ROS scavengers. The mitochondria-targeted ubiquinone derivative MitoQ is an established superoxide scavenger [65] and there is a single report of its ability to protect hearts from IR injury [66]. However, these data were obtained in a whole animal model in which rats were treated for 14 days with MitoQ in their drinking water before induction of myocardial ischemia. Thus, it cannot be ruled out that cardioprotection was secondary to longer term effects of MitoQ and not directly *via* mitochondrial ROS scavenging in the heart itself. Indeed, studies from this laboratory failed to demonstrate cardioprotection by MitoQ in the Langendorff-perfused heart [67]. Furthermore, in the many studies that have investigated the potential cardioprotective effects of a range of ROS scavengers during reperfusion, most have proved relatively ineffective at preventing IR injury in both the clinical setting and *in vivo* or *ex vivo* animal models of IR (reviewed in [68], [69]). Nevertheless, transgenic mice overexpressing either catalase or glutathione peroxidase are somewhat resistant to IR injury [70], [71].

The lack of effect of some ROS scavengers may be partially explained if ROS also play a role in cardioprotective signalling pathways as has been suggested [69], [72]. However, even in those cases where ROS scavengers do provide cardioprotection, it does not follow that this is the result of an inhibition of the initial phase of mPTP opening. Rather, it could be because of more efficient scavenging of the ROS produced after initial mPTP opening and thus the prevention of an escalating cascade of further ROS-sensitised mPTP opening and injury. This may also be true for the cardioprotection offered by pyruvate [73], [74], [75] and the anaesthetic propofol [76], [77], both of which are ROS scavengers. Pyruvate exerts especially powerful cardioprotection, but this is thought to be because it not only acts as a ROS scavenger but also maintains a more acid pH during ischemia and reperfusion [74], [75].

## 3 Assessing the evidence that succinate-driven reverse electron flow mediates superoxide production at Complex I during reperfusion

Complex I can generate large amounts of superoxide in the matrix by two mechanisms: first, at the flavin mononucleotide (FMN) site when it is in a highly reduced state such as under conditions of high proton motive force (pmf) and low rates of ATP synthesis (State 4); second, at another less well defined site when electrons are donated to the ubiquinone (CoQ) pool by REF. The latter occurs under conditions of very high pmf, especially when the pH gradient component is large, and is caused by the highly reduced state of the CoQ pool forcing electrons back from CoQH_2_ into Complex I, thus reducing NAD^+^ to NADH at the FMN site [78], [79]. Since the proportion of the FMN in the fully reduced state is set by the NADH/NAD^+^ ratio, the former mechanism produces more ROS in the presence of rotenone whereas the latter mechanism is blocked by rotenone. Importantly, superoxide production by either mechanism will be associated with a high matrix NADH/NAD^+^ ratio and this can be measured in the intact heart using surface fluorescence as can the redox state of flavoproteins which usually follows that of the NADH/NAD^+^ ratio [80], [81]. We have monitored the rapid changes in both the NADH/NAD^+^ and flavoprotein redox state as the heart is reperfused following ischemia using real time surface fluorescence and our data do not provide any support for a role of Complex I in superoxide production in the early phase of reperfusion [7].

### 3.1 Time course of Complex I oxidation

**Fig. 2:**
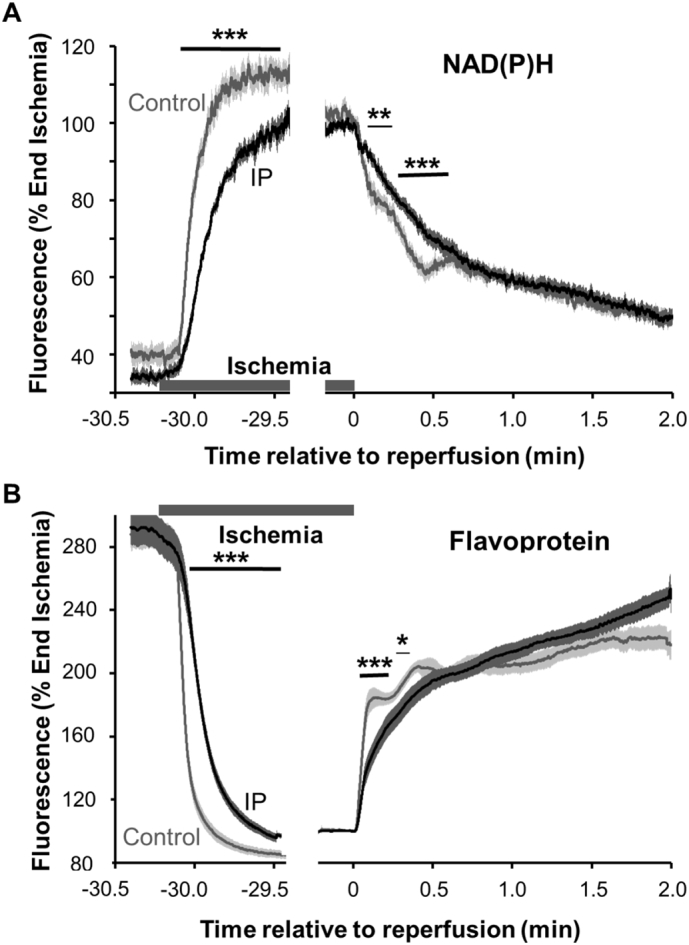
Time course of changes in flavoprotein and NAD(P)H surface fluorescence in the Langendorff-perfused heart subject to ischemia and reperfusion. Surface fluorescence data (normalised as % of values at the end of ischemia) are only shown for the start of ischemia (− 30 min) and reperfusion (0 min). Data are taken from [7] where further details may be found, and are presented as means ± SEM (error bars) of 7 control and IP hearts. Significant differences between control and IP hearts calculated by unpaired *t*-test are shown by horizontal lines (*p < 0.05; **p < 0.02, ***p < 0.01).

At the onset of ischemia, both NAD(P)H and flavoproteins rapidly become totally reduced as all oxygen is consumed, and they stay reduced until reperfusion is commenced when they rapidly re-oxidise as illustrated in Fig. 2.

In control and IP hearts re-oxidation of the flavoproteins was 50% complete within 5 and 15 s respectively and that of NAD(P)H at about 15 and 25 s respectively. The rapid re-oxidation is not unexpected since during ischemia ATP and creatine phosphate (CrP) are almost totally exhausted and are rapidly regenerated over the first few seconds of reperfusion [22], [82]. Indeed, oxygen consumption during the first 30 s of reperfusion is fast [7], consistent with the rapid oxidation of NADH required to fuel the increased rates of oxidative phosphorylation. However, several features of these data argue against significant superoxide production early in reperfusion. First, the highly reduced state of Complex I required for superoxide production is not maintained for more than a few seconds. Second, the pmf is unlikely to reach the high values associated with superoxide formation because of the rapid flow of protons back into the matrix through the F1Fo ATPase required for ATP re-synthesis. Third, the rate of re-oxidation of both flavoproteins and NAD(P)H is slower in IP hearts than control hearts. This does not readily fit with the proposal that IP reduces superoxide production from Complex I during early reperfusion as would be expected if superoxide is the key initial trigger for mPTP opening. This slower re-oxidation in IP hearts, and indeed the slower reduction during the onset of ischemia (Fig. 2), could reflect IP-mediated S-nitrosation of ND3 Cys39 and possibly other Complex I cysteines which inhibit its activity [24], [83], [84], [85], [86]. Another possible explanation would be that IP enhances inhibition of the mitochondrial ATP synthase by its inhibitor IF1 during ischemia, as has been observed in some studies (see [87]). This might lead to slower activation of oxidative phosphorylation and respiration during the first seconds of reperfusion. However, neither explanation would readily account for a reduction in matrix superoxide production by IP early in reperfusion.

### 3.2 Succinate measurements

**Fig. 3:**
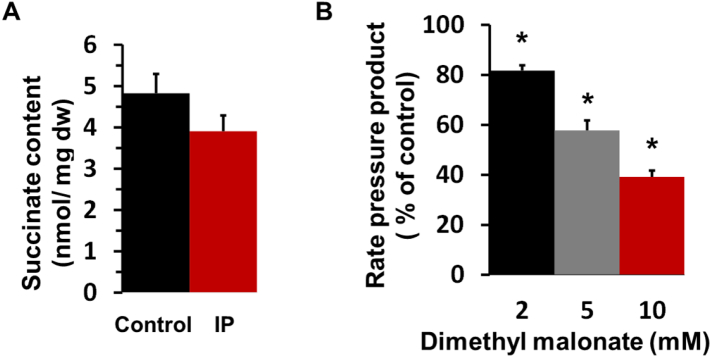
IP does not attenuate succinate accumulation in ischemia while dimethyl malonate impairs the hemodynamic performance of the Langendorff-perfused heart. Panel A shows the succinate content (nmol per mg dry weight; mean ± SEM, *n* = 6) of freeze clamped control (CI) and IP (IPI) hearts following 30 min global ischemia; values in normoxic hearts were below our limit of detection (< 0.5 nmol per mg). Data are taken from [7]. Panel B shows that the Rate Pressure Product (RPP) of normoxic Langendorff-perfused rat hearts was decreased by the presence of increasing concentrations of dimethyl malonate (DMM). Data are expressed as a percentage of values in the absence of DMM (29,900 ± 2800 mmHg*beat/min) and are given as means ± SEM (error bars) of 7 hearts. Statistical significance was determined by paired Student's *t*-test (*p < 0.01).

Chouchani et al. [23] confirmed earlier observations [88], [89] that succinate concentrations increase several-fold in ischemic hearts and rapidly decrease again on reperfusion. They proposed that this loss of succinate early in reperfusion reflects its oxidation by REF that drives ROS production at Complex I. As shown in Fig. 3A, we were able to confirm that tissue succinate levels increase many-fold following 30 min ischemia of Langendorff-perfused rat hearts, but importantly we found that a similar accumulation of succinate occurs in IP hearts during ischemia [7]. Since IP hearts exhibit less oxidative stress and mPTP opening after IR than control hearts [7], [15], [16], [52], [90], [91], this is not what might be anticipated if succinate oxidation early in reperfusion drives REF and consequent ROS formation at Complex I. It should also be noted that a study published nearly twenty years ago [92] demonstrated that perfusion of hearts with succinate protected them from IR injury rather than exacerbating injury as might be predicted if succinate plays an important role in early ROS formation. Furthermore, treatment of hearts with fumarate is also cardioprotective [93], [94] as is genetic knockdown of fumarate hydratase that causes fumarate accumulation of the heart [95], and both conditions are associated with increased succinate levels. In ischemia, fumarate and thus succinate are formed during the catabolism of aspartate and glutamate and this pathway can produce some ATP *via* succinate thiokinase mediated substrate level phosphorylation. The reduction of fumarate to succinate, mediated *via* NADH oxidation through Complex 1, ubiquinone and Complex 2 may also help maintain the mitochondrial pmf during ischemia. These two effects are probably responsible for some of the cardioprotection [93], [94], [95], [96]. Fumarate may exert an additional longer term effect through stabilization of the transcriptional regulator Nrf2, perhaps involving succination of its binding partner Keap1, that enhances the transcription of protective antioxidant genes [95].

**Fig. 4:**
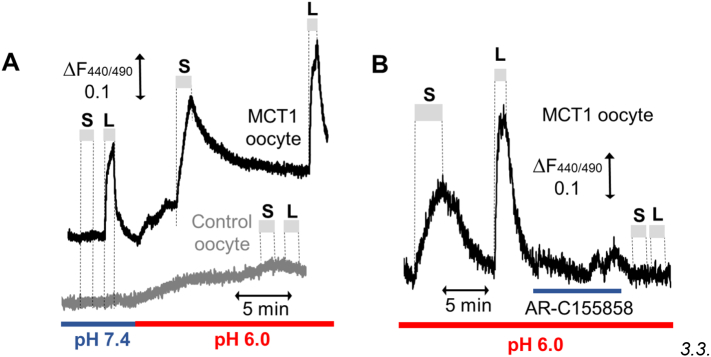
Succinate is transported by MCT1 as the monocarboxylate anion. MCT1 was expressed in *Xenopus laevis* oocytes proton-linked monocarboxylate transport measured in real time using the pH-sensitive fluorescent dye BCECF as described previously [100]. Panel A shows that 30 mmol/L l-lactate (L) is transported into and out of *Xenopus laevis* oocytes expressing MCT1 at both pH 7.4 and 6.0 but not control oocytes. By contrast, 30 mmol/L succinate (S) is only transported after the external pH has been decreased to 6.0 consistent with its transport as a monocarboxylate (*i.e.* in the monoprotonated form). Panel B shows that transport of both succinate and l-lactate at pH 6.0 is blocked by prior exposure of the oocyte to 1 μmol/L AR-C155858, a specific MCT1 inhibitor [100].

It should also be noted that the decrease in succinate observed during early reperfusion [23] was shown previously to be associated with its rapid efflux from the heart within 1–2 min of reperfusion [88] and therefore might not reflect its rapid oxidation as proposed by Chouchani et al. [23]. It is likely that the proton-linked monocarboxylate carrier, MCT1, which is highly expressed in the heart [97], facilitates this efflux of succinate since the intracellular pH drops to < 6.5 in the ischemic heart [98] and this will convert > 10% of succinate (pK_a2_ = 5.64) to its monocarboxylate form. It is known that short-chain substituted monocarboxylates are good substrates for MCT1 [99], and we have confirmed this to be the case for the monocarboxylate form of succinate by studying its uptake into *Xenopus laevis* oocytes expressing MCT1 as shown in Fig. 4. Significant proton-linked succinate transport was observed at pH 6.0, where 30% of succinate is in its monocarboxylate form, and this was blocked by AR-C155858, a specific and potent MCT1 inhibitor [100], whereas no detectable transport was observed at pH 7.4 where < 2% of succinate is present in its monocarboxylate form.

### 3.3 Cardioprotective effects of succinate dehydrogenase inhibition

Additional evidence provided by Chouchani et al. to support the importance of succinate oxidation for ROS production in early reperfusion was the demonstration that inhibition of succinate dehydrogenase (SDH) by malonate (generated from membrane-permeable dimethyl malonate – DMM) reduced ROS production in both isolated cardiomyocytes and hearts subject to IR [23]. In cardiomyocytes subjected to simulated IR the authors showed that DMM caused a significant decrease in ROS after 2 min “reperfusion”. However, they employed DHE as a ROS probe, which, as discussed earlier, is now recognised to be unreliable [30], [34], [35]. Moreover, as noted above, simulated IR in cardiomyocytes does not accurately reproduce the situation in the whole heart subject to IR. In an *in vivo* mouse model of reperfusion, Chouchani et al. showed that DMM decreased infarct size which confirms earlier studies that employed 3-nitropropionate [101], [102] and diazoxide [103], [104], [105], [106], [107] to inhibit succinate dehydrogenase. Although the authors used both mitoB and aconitase activity to show that DMM reduced matrix superoxide levels, these measurements were made after 15 min of reperfusion [23]. Thus, they may well reflect events occurring after mPTP opening and be secondary to cardioprotection mediated by DMM through other mechanisms. One such mechanism would be inhibition of flux through the citric acid cycle which utilises SDH as an essential component. This would lead to an impairment of both fatty acid and carbohydrate oxidation which might compromise the energy status of the heart. Indeed, as discussed more fully below, cardioprotection can be induced by a range of other agents and protocols that compromise the energy status of the heart, including a range of respiratory chain inhibitors [108], uncouplers [109] and transient ischemia on reperfusion (post-conditioning) [110]. Furthermore, as shown in Fig. 3B, we have demonstrated that in the Langendorff-perfused heart, treatment with DMM, at similar concentrations to those employed by Chouchani et al. [23], does lead to a progressive reduction in the rate pressure product (RPP) as the DMM concentration increased. This is entirely consistent with DMM mediating its cardioprotective effects through bioenergetic compromise.

### 3.4 Cardioprotective effects of Complex I inhibitors

Several studies have demonstrated that inhibiting Complex I during ischemia can reduce ROS formation and IR injury. This is the case for rotenone [111], amobarbital [108], [112], [113], [114], metformin [115], [116], [117], [118] and most recently by S-nitrosation of ND3 on Complex I mediated by the mitochondrial-targeted mitoSNO [24], [119]. It has been argued by Murphy, Kreig and colleagues that the cardioprotective effect of Complex I inhibition reflects inhibition of ROS production at Complex I [25], [26]. However, a range of other agents and protocols that compromise the energy status of the heart through diverse mechanisms have been shown to be cardioprotective. These include uncouplers of oxidative phosphorylation [109], [120], F_1_F_o_ ATP synthase inhibitors such as oligomycin and aurovertin [121], and the adenine nucleotide translocase (ANT) inhibitors atractyloside and bongkrekic acid [104], [105], [106], [107], [122], [123]. In addition, transient ischemia on reperfusion (known as post-conditioning) is a well-established cardioprotective protocol [110], [124] and is associated with impaired respiration and slower restoration of intracellular pH during reperfusion. In all these cases the heart is forced to generate ATP by glycolysis producing lactic acid which will result in a low intracellular pH being maintained for longer during the early stages of reperfusion. Opening of the mPTP is inhibited at pH values below 7 [5] and maintaining the mPTP in a closed state for longer during early reperfusion may allow restoration of ionic homeostasis, preventing calcium overload and thus an escalating cascade of mPTP opening, ROS production and injury so accounting for cardioprotection [1], [125].

## 4 Succinate stimulates ROS production following mPTP opening

**Fig. 5:**
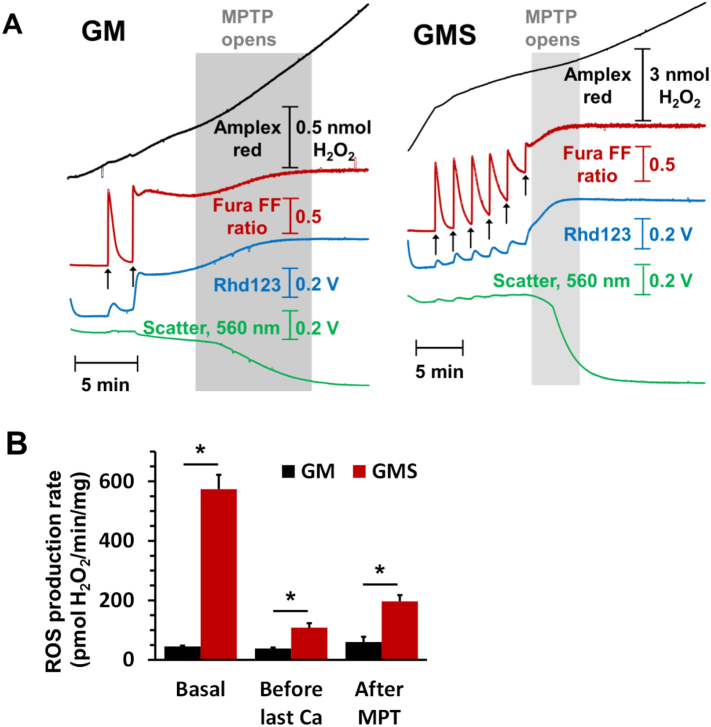
The [Ca^2+^]-sensitivity of mPTP opening in isolated heart mitochondria is reduced by succinate despite greater ROS production both before and after pore opening. Panel A shows the effects of sequential additions of Ca^2+^ (100 μmol/L) on isolated rat heart mitochondria incubated with 5 mmol/L l-glutamate + 2 mmol/L l-malate (GM) or GM plus 5 mmol/L succinate (GMS) on mPTP opening and ROS production. Opening of the mPTP was detected as a loss of Δψ (Rhd-123 fluorescence increase) or accumulated Ca^2+^ (Fura-FF fluorescence increase) and a decrease in LS while ROS production was monitored using Amplex Red in a parallel experiment on the same mitochondria. Further details may be found elsewhere [7]. Panel B presents mean rates of ROS production (± SEM of 6 different mitochondrial preparations) before Ca^2 +^ addition, after the penultimate Ca^2+^ addition and following pore opening.

As noted above, increased superoxide production can occur as a consequence of mPTP opening and this may lead to further mPTP opening in adjacent mitochondria [6], [7]. Thus an alternative explanation for the decreased ROS production caused by DMM treatment of both isolated cardiomyocytes subject to simulated IR and in the *in vivo* ischemic reperfused hearts [23] would be that succinate enhances ROS production after, rather than before, mPTP opening. In Fig. 5 we demonstrate that this is the case. Isolated heart mitochondria were energised with glutamate + malate in the presence (GMS) or absence (GM) of succinate and continuous measurements made of light scattering (LS), mitochondrial membrane potential (Δψ), extramitochondrial [Ca^2 +^] and production of hydrogen peroxide (rapidly formed from superoxide by SOD) as described previously [7]. As noted by others [126], [127], [128], hydrogen peroxide production before Ca^2+^ addition was greatly enhanced by succinate and this has been shown to involve superoxide production at both Complex I and SDH. Sequential additions of 100 μM Ca^2+^ were rapidly taken up by the mitochondria with a progressive reduction in both Δψ and the rate of hydrogen peroxide production until the mPTP opened (rapid and total loss of Δψ, release of accumulated Ca^2+^ and a large LS decrease). Hydrogen peroxide production then increased once more, confirming the increased superoxide production that occurs after mPTP opening. This may reflect the loss of cytochrome *c* and NADPH from the mitochondria, both of which are important for ROS scavenging [6], [22]. Importantly, even after mPTP opening, rates of hydrogen peroxide production were considerably greater when succinate was present than in its absence as shown in Fig. 5B. Very recent data from Weiss's laboratory have also confirmed that succinate increases ROS production in heart mitochondria following mPTP opening [129]. This ROS was shown to be produced primarily at Complex II but might also involve Complex III if this were damaged during ischemia [130].

Another important aspect of the data we present in Fig. 5A is that many more additions of Ca^2+^ were required to induce mPTP opening in the presence of succinate than its absence, despite succinate inducing much higher rates of hydrogen peroxide production. These data are in agreement with those obtained by others using skeletal muscle mitochondria [131] and suggest that succinate oxidation may actually protect against mPTP opening despite the greater ROS production. This casts further doubt on the proposal of Chouchani et al. [23] that succinate induced ROS production is a critical mediator of IR injury but is supportive of earlier studies in which succinate was found to be cardioprotective [92].

## 5 Alternative triggers for mPTP opening early in reperfusion

Our review of the available evidence leads us to conclude that it is unlikely that an increase in matrix superoxide production at Complex I, driven by REF from succinate, is the critical trigger for mPTP opening in the initial phase of reperfusion. Rather, we suggest that the majority of ROS are produced later in reperfusion and that this occurs as a consequence of mPTP opening. This helps to drive a progressive cascade of damage to the cardiomyocytes, including further mPTP opening and ROS production as well as damage to proteins involved in ionic homeostasis and calcium handling [132]. The result is an expanding area of necrotic cell death that forms the infarct. If this conclusion is correct then there must be other factors that trigger the initial mPTP opening and that can be attenuated by cardioprotective protocols such as IP. We consider some of these below.

### 5.1 The role of Ca^2 +^

It is well established that calcium acts as an essential trigger for mPTP opening and that many other factors that enhance pore opening do so by increasing its sensitivity to [Ca^2+^] [1], [12]. Increases in matrix [Ca^2+^] have been demonstrated previously in both cardiac myocytes [133] and perfused hearts [134] subject to ischemia and reperfusion. We have recently confirmed that increases in mitochondrial [Ca^2+^] can be detected in the Langendorff-perfused heart after 30 min ischemia and 90 s of reperfusion [7]. It seems probable that this increase in matrix [Ca^2+^] is important for mPTP opening since hearts of mice with an adult cardiomyocyte-specific deletion of the mitochondrial calcium uniporter (MCU) were shown to exhibit reduced IR injury [135], [136]. These data confirmed earlier reports that the MCU inhibitors ruthenium red and Ru360 are cardioprotective [137], [138]. Furthermore, mice with heart-specific deletion of the mitochondrial Na^+^/Ca^2+^ exchanger (NCLX), which is responsible for most Ca^2+^ efflux from the matrix of heart mitochondria, experienced substantial mitochondrial Ca^2+^ overload. This was accompanied by increased generation of superoxide and necrotic cell death in the heart and led to death of the mice within a few days. However, the mice survived considerably longer if mitochondrial permeability transition pore activation was attenuated by additional knockout of Cyp-D. Conversely, overexpression of NCLX in the mouse heart enhanced mitochondrial Ca^2+^ efflux, prevented opening of the mPTP and protected against ischemia-induced cardiomyocyte necrosis and heart failure [139].

These data all confirm the importance of increased matrix [Ca^2+^] in triggering the mPTP opening that leads to increased ROS production and damage to the heart. However, we found no evidence that the cardioprotection afforded by IP was accompanied by any attenuation of this early rise in matrix [Ca^2+^], although IP did prevent the calcium overload that occurred later in reperfusion [7]. We confirmed that this later calcium overload was secondary to mPTP opening by demonstrating that it was prevented by pre-treatment of the hearts with CsA, a well-established mPTP inhibitor [7]. Others have come to a similar conclusion using an isolated myocyte model of IR [140]. It seems likely that the calcium overload induced by the initial phase of mPTP opening may act in conjunction with the parallel increases in ROS to cause additional mPTP opening in adjacent “unopened” mitochondria. This may mediate a progressive cascade of further ROS production and mPTP opening that will lead to a spreading area of necrotic cell death to form the infarct as discussed above. Indeed, waves of [Ca^2+^] and ROS that precede mPTP opening have been observed by confocal microscopy in the intact contraction-inhibited heart subject to hypoxia and re-oxygenation [141].

Overall, we conclude that, although IP attenuated the secondary calcium overload that follows mPTP opening, consistent with its ability to inhibit mPTP opening [7], [52], [90], [91], its inability to prevent the increase in matrix [Ca^2+^] that occurs in the first 90 s of reperfusion [7] implies that another factor must play a critical role in regulating this first phase of mPTP opening. One possible candidate is mitochondria-bound hexokinase 2 (HK2) whose extent of dissociation from mitochondria during ischemia correlates with infarct size and is prevented by IP [27], [28], [29].

### 5.2 The role of mitochondria-bound hexokinase 2

**Fig. 6:**
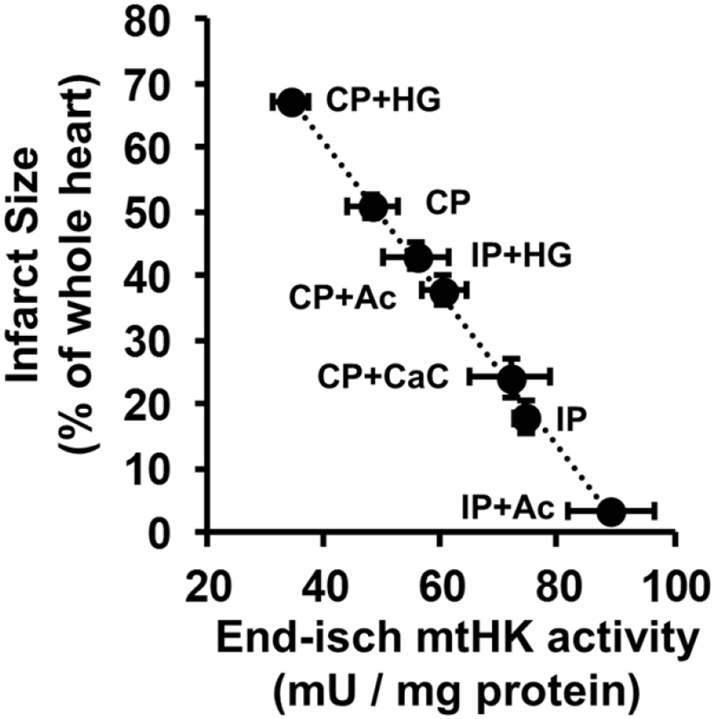
The extent of HK2 dissociation from mitochondria during ischemia is strongly correlated with infarct size following reperfusion. HK activity was measured in mitochondria isolated after 30 min ischemia and the infarct size after 2 h reperfusion. The changes in HK activity was confirmed to be due exclusively to HK2 dissociation by Western blotting. The pre-treatments used to modify HK2 binding at the end of ischemia were: Control (CP), ischemic preconditioned (IP) and perfusion with acetate (Ac), high [Ca^2+^] (CaC) or high glucose (HG) with or without IP. Data are taken from [22].

It has been known for many years that both hexokinase 1 (HK1) and hexokinase 2 (HK2) can bind to mitochondria and that such binding may play an important role in the regulation of hexokinase activity and thus of glucose metabolism [27], [142], [143]. However, more recently evidence has emerged that binding of these enzymes to the outer mitochondrial membrane (OMM) may also decrease apoptosis mediated by members of the Bcl2 family and antagonise mPTP opening [28]. Cardiac myocytes express both HK1 and HK2 and both bind to the OMM through an N-terminal hydrophobic domain but a significant proportion of HK2 is present in the cytosol [144], [145]. The extent of HK2 binding to mitochondria depends on the prevailing metabolic conditions with high concentrations of glucose-6-phosphate (G-6-P) favouring HK2 dissociation [143], [146]. Zuurbier and colleagues demonstrated a progressive loss of bound mitochondria-bound HK2 during ischemia of the heart [147] and we confirmed this, showing substantial loss of mitochondrial HK2, but not HK1, after 30 min global ischemia [148]. This was associated with a loss of cytochrome *c* from mitochondria suggesting enhanced permeability of the OMM. Furthermore, IP prevented both the dissociation of HK2 and the loss of cytochrome *c* [22] and also decreased the calcium sensitivity of mPTP by isolated mitochondria [16]. By manipulating the metabolism of the perfused heart prior to ischemia we were able to demonstrate [22] that there was a very strong inverse correlation between the amount of HK2 remaining bound to mitochondria at the end of ischemia and the infarct size after 120 min of reperfusion as shown in Fig. 6.

We have also shown that the increased susceptibility to IR of hearts from high fat fed mice is associated with less mitochondrial bound HK2 [149] while others have demonstrated that hearts from HK2^+/−^ mice have reduced HK2 content and are more sensitive to IR injury [150]. As such HK2 binding to mitochondria behaves in a manner that would be expected of a key factor involved in determining the extent of mPTP opening and IR injury. Thus, HK2 dissociation from mitochondria during ischemia will enhance cytochrome *c* release and predispose the mPTP to open early in reperfusion under conditions of increased matrix [Ca^2+^]. By preventing this HK2 dissociation from mitochondria during ischemia, IP prevents both cytochrome *c* loss and mPTP sensitisation thus accounting, at least in part, for its cardioprotective effect. Very recent data from Zuurbier's laboratory have provided further evidence in support of this hypothesis. Rat hearts were perfused with low concentrations (200 nM) of a membrane permeable peptide (TAT-HKII) that dissociates bound HK2 from mitochondria and which was without significant effect on normoxic hearts. However, after 15 min ischemia, when reperfusion of control hearts caused only reversible injury (impaired hemodynamic function), the presence of the TAT-HKII peptide caused irreversible injury (lactate dehydrogenase release) [151].

Possible mechanisms by which IP may attenuate HK2 dissociation from mitochondria during ischemia have been comprehensively reviewed elsewhere [28] and are only briefly summarised here. Attenuation of the ischemia-induced increase in [G-6-P] and decrease intracellular pH are critical, and reflect decreased glycogen levels prior to ischemia [152], [153], [154]. Additional signalling pathways have also been proposed including translocation of PKCε to the mitochondria where it may phosphorylate VDAC1 to increase HK2 binding [155], although we were not able to confirm this [16]. GSK3β and Akt have also been implicated in IP [156], [157]. Indeed, it has been proposed that the mechanisms of many cardioprotective regimes converge to phosphorylate and inhibit GSK3β whose activity promotes mPTP opening [158], [159]. Interestingly, Akt has also been reported to phosphorylate Thr^473^ of HK2 directly with the result that its binding to mitochondria in cardiomycoytes is enhanced while its sensitivity to dissociation by increased concentrations of G-6-P is reduced [160], [161]. However, others have reported that neither Akt stimulation with insulin nor genetic ablation of VDAC isoforms 1 and 3 affect cell death induction by HK2 dissociation [144]. Furthermore, insulin, a potent activator of the Akt pathway, is not cardioprotective unless added during reperfusion [158]. Indeed, we found that pre-ischemic treatment of hearts with insulin in place of the IP protocol caused Akt and GSK3β phosphorylation to increase several-fold yet no cardioprotection was observed [16] while others have reported that such treatment may even suppress cardioprotection by IP [162]. Nor were we able to detect significant IP-mediated changes in phosphorylation of any mitochondrial proteins (including VDAC and HK2) [16]. Whatever the exact mechanisms(s) underlying the regulation of HK2 binding to mitochondria, the very strong inverse correlation between the extent of its binding at the end of ischemia with the extent of damage (infarct size) following reperfusion (Fig. 6) suggests that it may play a key role in cardioprotection. An attractive feature of this proposal is that it provides a plausible explanation of how, by converging on HK2 binding to mitochondria, a diverse range of known cardioprotective protocols with distinct signalling pathways can inhibit mPTP opening on reperfusion and so reduce IR injury.

### 5.3 The role of mitochondrial contact sites and cristae morphology

The mechanism(s) that link(s) HK2 binding to mPTP opening remain uncertain, but an attractive hypothesis for which there is increasing evidence is that HK2 binding modulates the interaction between the inner mitochondrial membrane (IMM) and the OMM. Dissociation of HK2 is proposed to alter the mitochondrial morphology and cristae structure in such a way as to increase OMM permeability to cytochrome *c* and sensitise mPTP opening to matrix [Ca^2+^] [28]. The presence of “contact sites” between the IMM and OMM, where the two membranes interact closely, was first revealed by freeze-fracture of mitochondria and were shown to be dynamic structures which are affected by the metabolic status of mitochondria [163], [164]. Several proteins have been implicated in their formation including VDAC, ANT and, when present, HK1, HK2 and creatine kinase (CK) [164], [165]. Originally thought to function primarily to enhance the transfer of ATP from the matrix to the cytosol [163], contact sites were subsequently implicated in the regulation of OMM permeability and mPTP opening [166]. We presented evidence that destabilisation of contact sites may facilitate opening of cristae junctions and thus access of cytochrome *c* within the cristae to the permeation pathways in the OMM, and also increase the sensitivity of the mPTP towards [Ca^2+^] [167]. HK2 binding stabilises contact sites [168], [169] and thus it would be predicted that the loss of HK2 during ischemia might lead to contact site breakage. In the perfused heart, there is good evidence that this is the case since operation of the creatine phosphate (CrP) shuttle, which involves the interaction of the ANT, CK and VDAC at contact sites, is impaired following ischemia [170] but less so following IP [22], [171]. Furthermore, using a variety of preconditioning stimuli to modify mitochondrial HK2 binding at the end of ischemia we confirmed that maintenance of contact sites during ischemia, as reflected in the recovery of CrP and hemodynamic function on reperfusion, was greatest in hearts with the highest mitochondrial HK2 binding [22]. The importance of contact sites in protecting mitochondria from mPTP opening is also consistent with the observation that knockout of CK, whose presence stabilises contact sites [172], makes hearts more sensitive to IR injury [173]. In addition, liver mitochondria from mice genetically modified to express mitochondrial CK (which is normally absent in liver) demonstrated a 3-fold increase in the number of contact sites as observed by EM [172], and were more resistant to mPTP opening by calcium [174].

Bcl-2 family members may also associate with the OMM at contact sites, possibly binding to VDAC [142], [175], [176], [177]. These proteins can influence both OMM permeability and mPTP activity (see [178], [179], [180]) and Bcl-xL has been shown to be lost from heart mitochondria during ischemia, perhaps leading to unmasking of pro-apoptotic members of the Bcl-2 family such as Bax and Bak that are already in the OMM [22]. Furthermore, adenovirus-mediated Bcl-xL gene transfer has been reported to protect hearts from IR injury [181] while hearts from mice in which both Bax and Bak have been knocked out are also protected from IR [182]. The activity of members of the Bcl-2 family can also be regulated by phosphorylation, as well as by proteolysis and translocation (see [178], [179], [180]) and this may provide yet further mechanisms by which preconditioning can attenuate OMM permeabilisation and mPTP opening in conjunction with its enhancement of HK2 binding. So too may the HK2-regulated oligomerisation of VDAC1 in the OMM that has recently been proposed as a pathway for cytochrome *c* release in apoptosis [176], [183]. Indeed, inhibitors of this process have been developed that prevent selenite-induced apoptosis and the associated cytochrome *c* release, ROS formation, mitochondrial depolarization and calcium overload [184]. Significantly this is accompanied by prevention of HK dissociation from the mitochondria and the authors propose that HK binding to VDAC1 prevents its oligomerization. This provides another plausible explanation for the relationship between mitochondrial HK binding and resistance to apoptosis and mPTP opening.

### 5.4 The role of mitochondrial fission

There may also be a link between contact site stability, mitochondrial cristae morphology and mitochondrial fission [28]. Thus the release of cytochrome *c* from the intermembrane space during apoptosis, which is associated with mitochondrial fission, involves changes in mitochondrial cristae morphology allowing the cytochrome *c* within the cristae to gain access to the permeabilised OMM [180]. A key player in mitochondrial fission is the GTPase Dynamin-related protein 1(Drp1) [180], [185] which must be recruited to the OMM. Importantly, mitochondria have been reported to undergo fission during ischemia of the perfused heart and this might be a critical factor in inducing the observed cytochrome *c* release [186]. Furthermore, inhibiting Drp1 activity with the inhibitor Mdiv-1 [186], [187], [188], [189] or transgenic expression of a dominant negative form of Drp1 [190] was found to reduce infarct size following IR. This was accompanied by preserved mitochondrial morphology, reduced mitochondrial ROS production and improved hemodynamic function [189]. Another attractive feature of Drp1 is the complex regulation of its translocation to and from mitochondria. Drp1 can be phosphorylated on either Ser^616^ or Ser^637^ by different protein kinases but only the former promotes its translocation to mitochondria [189]. Interestingly, the kinase responsible for Ser^616^ phosphorylation is PKCδ which becomes active during ischemia [191]. Similarly, Ser^637^ is phosphorylated by PKA that is active after pre-conditioning or TP [21] and promotes Drp1 dissociation from mitochondria. Thus, in a similar manner to HK2, it would seem possible that Drp1 can also act as a common locus that links different responses to IR and mitochondrial fate. Furthermore, Drp1 has been shown to associate with mitochondria during ischemia [187], [192], [193] and to facilitate calcium flux and cristae remodeling during apoptosis [194] in an Mid49/51-dependent and OPA1-independent fashion [195]. It is possible that the opposite translocation of Drp1 and HK2 during ischemia and cardioprotective regimens may reflect a shared but competing binding region on the OMM. Indeed, very recent studies in neonatal cardiac myocytes have demonstrated a reciprocal interaction between Drp1 and HK2 binding to mitochondria [196]. Treatment of the myocytes with 100 μM palmitic acid for 2 h enhanced Drp1 binding to mitochondria while reducing HK2 binding, and this was associated with increased mPTP opening and ROS and [Ca^2+^] levels. Treatment with 2-deoxy-glucose also reduced mitochondrial HK2 binding while promoting Drp1 translocation to mitochondria. Furthermore, knockdown of Drp1 using siRNA prevented the loss of HK2 induced by palmitic acid as did increasing Drp1 phosphorylation by Akt activation which also prevented the mPTP opening and the associated increases in [ROS] and [Ca^2+^].

## 6 Conclusions

**Fig. 7:**
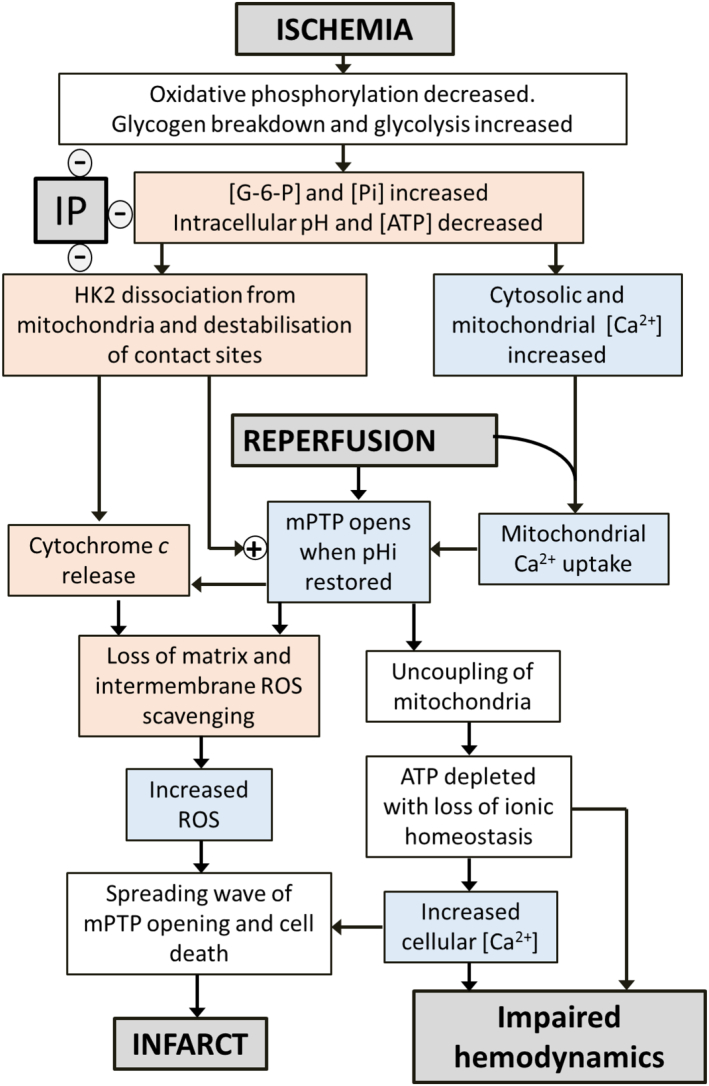
Proposed sequence of events in ischemia and reperfusion that lead to mPTP opening and reperfusion injury and their attenuation by IP. This scheme is a slightly modified version of those presented previously [7], [28].

Our own data together with a critical analysis of the published data of others lead us to conclude that it is unlikely that significant superoxide production in the mitochondrial matrix, driven by succinate-fuelled REF, occurs in the early phase of reperfusion (*i.e.* the first 2 min). Hence we suggest that this mechanism is unlikely to be a critical mediator of IR injury. Rather we would argue that the evidence favours the initial phase of mPTP opening being triggered by factors other than matrix ROS while the large increase in ROS seen later in reperfusion occurs as a consequence of mPTP opening. This secondary rise in ROS may play an important role in causing an escalating cascade of mPTP opening that leads to a spreading wave of necrotic cell death and the formation of the infarct. An important stimulus for the first phase of mPTP opening is likely to be the observed increase in mitochondrial matrix [Ca^2+^] during ischemia and early reperfusion. However, it cannot be the only trigger because the increase in [Ca^2+^] is not attenuated by IP, yet IP inhibits mPTP opening and provides cardioprotection. A promising additional candidate for regulating the initial phase of mPTP opening is mitochondrial bound HK2 which dissociates from the mitochondria during ischemia, but not following IP. Dissociation of bound HK2 is associated with an increase loss of cytochrome *c* from the mitochondria during ischemia and an enhanced sensitivity to mPTP opening. This provides an explanation of the very close correlation between the extent of HK2 loss from mitochondria during ischemia and the extent of injury (infarct size) on subsequent reperfusion. The mechanisms linking HK2 dissociation to OMM permeabilisation, cytochrome *c* release and mPTP sensitisation remain to be fully established but the available evidence implicates several related processes: the stability of contact sites between the inner and outer membranes, cristae morphology and VDAC1 oligomerisation. This is illustrated diagrammatically in Fig. 7. The involvement of these interacting processes provides potential loci for the established effects on IR injury of mitochondrial fission proteins such as Drp1 (knockout/inhibition is cardioprotective), members of the Bcl-2 family such as Bcl-xL, whose overexpression is cardioprotective, and Bax and Bak whose knockout is cardioprotective, and proteins enhancing contact site formation such as HK2 and mitochondrial CK whose knockout enhances damage. However, further work will be required to test this hypothesis and elucidate the underlying mechanisms that link these processes to the regulation of mPTP opening and the development of IR injury.

## Sources of funding

Medical Research Council G0801237.

British Heart Foundation RG/08/001/24717 and PG/12/40/29634.

## Disclosures

None.
